# Conversion of Phase Information into a Spike-Count Code by Bursting Neurons

**DOI:** 10.1371/journal.pone.0009669

**Published:** 2010-03-12

**Authors:** Inés Samengo, Marcelo A. Montemurro

**Affiliations:** 1 Centro Atómico Bariloche and Instituto Balseiro, San Carlos de Bariloche, Argentina; 2 Faculty of Life Sciences, The University of Manchester, Manchester, United Kingdom; Cuban Neuroscience Center, Cuba

## Abstract

Single neurons in the cerebral cortex are immersed in a fluctuating electric field, the local field potential (LFP), which mainly originates from synchronous synaptic input into the local neural neighborhood. As shown by recent studies in visual and auditory cortices, the angular phase of the LFP at the time of spike generation adds significant extra information about the external world, beyond the one contained in the firing rate alone. However, no biologically plausible mechanism has yet been suggested that allows downstream neurons to infer the phase of the LFP at the soma of their pre-synaptic afferents. Therefore, so far there is no evidence that the nervous system can process phase information. Here we study a model of a bursting pyramidal neuron, driven by a time-dependent stimulus. We show that the number of spikes per burst varies systematically with the phase of the fluctuating input at the time of burst onset. The mapping between input phase and number of spikes per burst is a robust response feature for a broad range of stimulus statistics. Our results suggest that cortical bursting neurons could play a crucial role in translating LFP phase information into an easily decodable spike count code.

## Introduction

**Figure 1:**
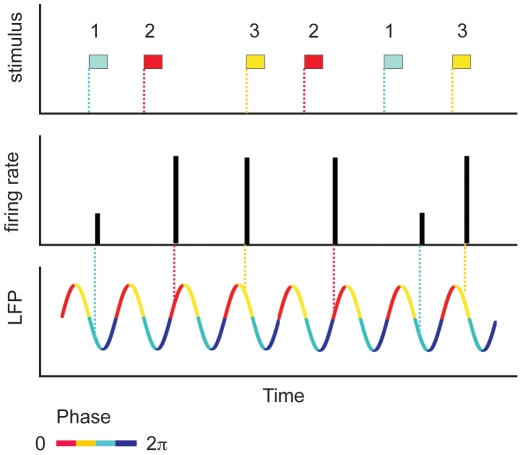
Schematic representation of a phase code. Firing rate of a cell in response to three different stimuli. By reading out the number of spikes per unit time (the height of the bars), stimulus 1 is distinguishable from the other two stimuli. However, stimuli 2 and 3 induce the same response, and therefore cannot be discriminated on the basis of the firing rate alone. However, if also the timing with respect to the phase of the LFP is taken into account, stimuli 2 and 3 become distinguishable. Inspired on a figure from [11].

Cortical networks have a rich repertoire of rhythmic oscillations [1]. Previous studies [2], [3] have suggested that coherent oscillations could be used in the brain as an effective time frame regulating neural coding. For example, in hippocampal place cells, the firing of a place cell indicates that the animal is inside the place field, and thereby provides coarse location information [4]. More detailed information about the precise position inside the place field can be obtained from the phase of the theta rhythm at burst onset [5], [6]. More generally, in several brain areas, the relative timing between the firing onset of pyramidal neurons and the local field potential (LFP) encodes additional information about the external stimulus, not present in spike counts alone. Although there have been suggestions that similar mechanisms could operate at the neocortical level [7], [8], direct quantitative evidence using information theoretic analysis of in-vivo data became available only recently for visual [9] and auditory [10] cortices. Those studies showed that when the timing of spikes is measured relative to the phase of the LFP, there is a significant increase in information about the stimulus carried by the spike train. The advantages of a phase-of-firing encoding are illustrated in Figure 1 [11]. The bars represent the firing rate of a cell in response to three different stimuli. Based on the traditional view that information is encoded in the mean firing rate, stimulus 1 can be discriminated from the other two stimuli, since it generates a weaker response. However, both stimuli 2 and 3 give rise to the same firing rate, and therefore cannot be discriminated using the firing rate alone. However, if the relative timing between firing onset and the phase of the ongoing LFP oscillation is also taken into account (phase-of-firing code), then the responses to stimuli 2 and 3 become distinguishable, since they occur at different phases of the LFP. In the figure we used a color code to represent the phase of the LFP in sections of π/2. The phase-of-firing code increased the information transmitted by cortical cells by around 54% in visual cortex [9] and by more than 100% in auditory cortex [10], when compared to the information conveyed by the spike rate alone. However, it is still unclear how phase information could be read out by distal downstream target cells, since the LFP at the soma of the pre-synaptic afferents is not directly accessible to remote neurons. The aim of this work is to show that cortical bursting neurons can translate phase information into a spike-count format, thus making it available to other brain regions.

The ultimate origin of the LFP is still a matter of debate. One line of thought supports the idea that the LFP is generated by a weighted linear sum of the membrane potentials of the neurons in a local neighborhood [12]. Since the membrane potential of spiking neurons is an oscillating variable per se, in this view, the LFP could be understood as a mean field variable describing the collective dynamics of an ensemble of coupled non-linear oscillators [13]–[15]. The LFP thus results as a measure of the coherence of the spiking activity in a given local area. There is, however, an alternative line of thought based on experiments performed along several decades [16]–[23]. This line proposes that the LFP results from the average synaptic and dendritic processes reaching a given cortical neighborhood [24], [25]. Thus, the LFP mainly reflect coherent input into the region, instead of coherent output, as advocated by the previous view [26]. Therefore, pyramidal neurons in cortex are presumably driven by time-varying signals whose temporal evolution unfolds similarly to that of the LFP [27]. When the majority of the synaptic inputs to a given area come from local recurrent connections, the two views coincide. However, in those brain areas that receive massive input from distal areas, the two views make different predictions for the LFP. In this paper, we adopt this second point of view, and see the LFP as an input signal driving the modeled neuron [26].

In order to explore the phase code instantiated by bursting pyramidal neurons, we simulated a widely used two-compartment neural model driven by different types of time-dependent input currents. We characterized the way the stimulus phase is represented in the output spike trains, and compared the performance of the phase code with other alternative codes. We conclude that the number of spikes in each burst provides a robust representation of input phase. Therefore, by reading out the intra-burst spike count, a downstream neuron can extract information about the temporal properties of the input current exciting the neuron. Our analysis thus presents a biologically plausible mechanism capable of translating phase information into a spike count code.

## Results

**Figure 2:**
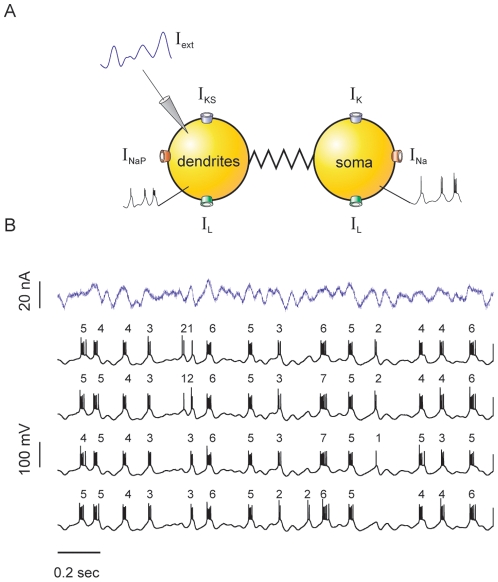
Pyramidal neuron model. (A) Schematic representation of the two-compartment model and the ionic currents involved. The stimulus is injected into the dendritic compartment. Next to each compartment we also show an example trace of the membrane potential during burst generation. The ionic currents associated with each compartment are also indicated. See Methods for mathematical details. (B) Typical responses obtained for a random input current. The top trace represents a sample stochastic stimulus. The stimulus consists of a signal part (low-pass filtered Gaussian white- noise with 10 Hz cut-off frequency), and a noise component (low-pass filtered Gaussian white- noise with 1 KHz cut-off frequency, whose standard deviation (SD) is equal to ¼ of the SD of the signal). The four example traces correspond to the output of the neuron when stimulated with the same signal component, and four different realizations of the noise. The numbers on top of the traces indicate the number of spikes in the bursts.

We simulated the activity of a bursting model neuron (see Methods and Figure 2A) driven by a time-dependent signal that is proportional to the LFP. This driving signal will be henceforth called the *stimulus*. Example burst-like responses are displayed in Figure 2B. In each single trial, bursts appear with variable duration: some of them are short, containing just one or two spikes (*n* is equal to 1, or 2), whereas others are long enough to comprise up to 7 spikes (*n* = 7). In the subsequent traces of Figure 2B, we show the response of the model cell to repeated presentations of the stimulus, subjected to input noise that is drawn independently in each stimulus presentation. Even though the standard deviation of the noise is as high as one quarter of the standard deviation of the signal, the number of spikes per burst *n* remains fairly constant throughout different stimulus presentations. Hence, *n* displays a remarkable flexibility within each trial, though little variation is observed across trials. This means that the number of spikes in each burst must encode information about some specific stimulus feature. The aim of our work is to reveal this feature.

### Response to constant stimuli

**Figure 3:**
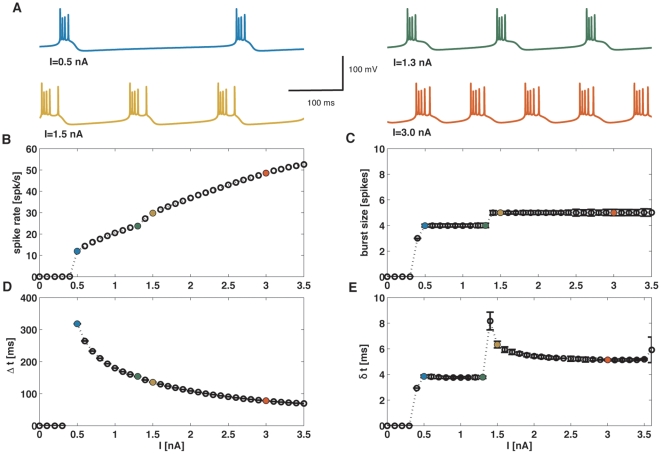
Response to a constant input current. (A). Membrane potential traces for different intensities of the input current. In the lower panels (B–D), the colored symbols correspond to the traces in A of matching colour. (B) Spike firing rate as a function of the input current intensity. The two singular points correspond to the onset of firing at 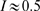 nA, and the change in the size of bursts from 3 spike bursts to 4 spike bursts at 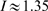 nA. (C) Burst size (in number of spikes per burst, *n*) as a function of the input current. (D) Inter-burst time interval Δt. Apart from the discontinuity at the onset of firing, the interval between bursts decreases smoothly as the input current increases. (E) Intra-burst inter-spike interval δt. After a rapid adjustment following the singular points corresponding to firing onset and burst size transition, the intra-burst ISI remains essentially unaffected by variations on the constant input.

As a first step, we considered constant input currents, which are useful to motivate the study of more natural signals (see below). When driven with a constant stimulus, after an initial transient period model neurons set onto a periodic firing regime (Figure 3A). The mean firing rate, the intra-burst period and the inter-spike interval within each burst depend on the intensity of the input current, as explained in the supporting Text S1 and Figure 3B, D and E. The number of spikes per burst *n*, however, changes much more slowly as the input current is varied. For instance, while the firing rate varies from around 30 Hz to 50 Hz, the number of spikes per burst remains fixed at 5. This rigid behavior of the burst size contrasts with the flexibility observed in Figure 2B, where a broad variation in *n*-values is observed. The wider range of burst sizes obtained with time-dependent stimuli suggests that *n* encodes dynamic stimulus features. To explore this hypothesis in a systematic way, in the following sections we used time-varying stimuli of increasing complexity.

### Sinusoidal stimulation

**Figure 4:**
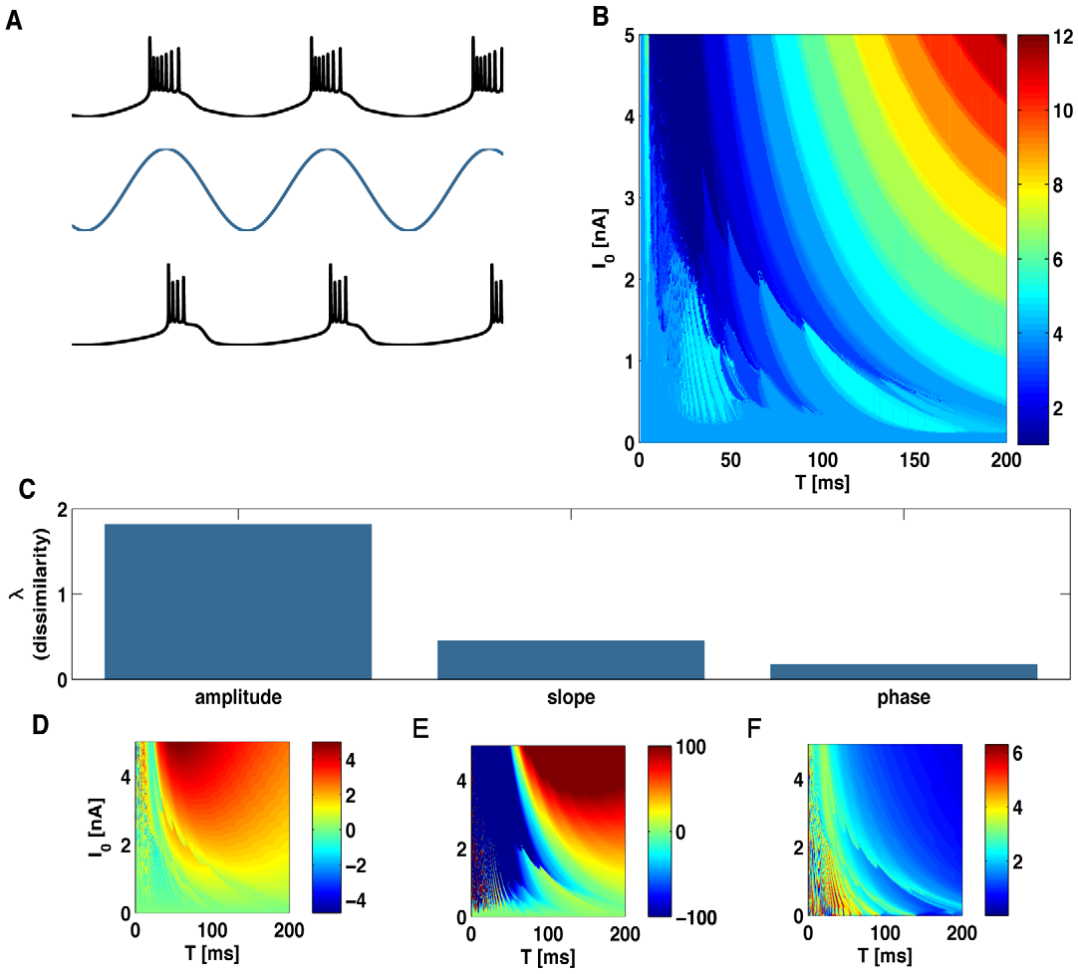
Response to sinusoidal input. (A) Sample membrane potential traces (black) for two different stimuli (blue, not to scale) differing in their amplitude. Burst generation locks to the stimulus. (B) Color map of the average burst size as a function of both the maximum amplitude, 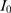, and period, 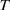, of the periodic stimulus. (C). Dissimilarity index (see Methods) for three candidate burst codes representing the amplitude, the slope, and the phase of the input signal at the time of burst initiation. The index quantifies the difference between the level lines in the color maps in panels D–F and those of the burst-size map in B. The dissimilarity index is minimal for the phase code. (D–F). Average value of the stimulus amplitude (D), slope (E) and phase (F) at burst onset, as a function of the maximum stimulus amplitude, 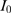, and period 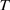.

When stimulated with low-frequency sinusoidal currents, the neuron locks to the input oscillations, firing one burst per stimulus cycle, as shown in Figure 4A. As the input frequency increases, more complex patterns are observed: some stimulus cycles are missed, bursts are not necessarily equally spaced, and they contain a variable number of spikes (see examples in supporting Figure S1A). Such complex responses appear because high-frequency stimulation of non-linear oscillators can give rise to erratic behavior, characterized by chaotic traces [28]–[30]. Therefore, in this study we restrict the analysis to fairly slow input currents, thus setting the basis for a candidate neural code.

As opposed to the rigid behavior in response to constant stimuli, the number of spikes per burst *n* is highly sensitive to the stimulus period and amplitude (Figure 4B). Thus, the temporal structure of the signal has a profound effect in the internal composition of bursts. Rapid or shallow stimuli generate bursts with only a few spikes, whereas slow or strong oscillations elicit long bursts. The dependence of the firing rate of the cell with the stimulus amplitude and frequency is discussed in the supporting Text S2 (see also the supporting Figure S1B).

Which stimulus attribute is best represented by the number of spikes per burst? This question may be phrased more precisely by asking which is the stimulus attribute that, at the time of burst onset, is most tightly related to *n*. When two quantities co-vary, they have the same contour lines [31], [32]. Hence, we need to decide which stimulus attribute has the same contour lines as the ones corresponding to *n*. To assess this issue, we defined a coefficient of dissimilarity 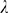 (see *Methods*) that quantifies the difference between the contour lines in Figure 4B, and the ones corresponding to the three different stimulus attributes of Figure 4D, E and F.

This coefficient is a variance measure, quantifying the variability of *n* within each contour line of the candidate stimulus feature. If λ = 0, the two quantities co-vary perfectly. Larger values of λ indicate a weaker correspondence between them. For fast input stimuli, a quantitative assessment of the co-variation of *n* with different stimulus features becomes unreliable, since the contour lines of all quantities become highly complex, with structures that are repeatedly nested one inside the other. In particular, in coincidence with the behavior of other non-linear oscillators subjected to forcing oscillatory input, in the high frequency region the neuron model exhibits a series of bands characterized by chaotic dynamics interleaved with windows of periodicity (see references [28]–[30]). Any numerical procedure to detect contour lines becomes discontinuous in this range. Therefore, all calculations of 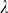 are performed within the range of *T* from 50 to 200ms. In Figure 4C, we show the dissimilarity index for the three level plots of Figure 4D–F, associated to the stimulus amplitude (D), slope (E) and phase (F) at burst onset.

As a first option, we considered the possibility that *n* represented the stimulus amplitude at burst onset (Figure 4D). The coefficient of dissimilarity between *n* and the amplitude is 1.81. As a second option, and following Kepecs *et al.* [33] and Kepecs and Lisman [34], we evaluated the co-variation between *n* and the stimulus slope at burst onset (Figure 4E). The degree of dissimilarity between these two quantities is 0.45, implying a significant improvement with respect to the amplitude.

In this paper, we put forward a novel alternative, namely, that the number of spikes per burst represents the phase of the stimulus at burst onset. In Figure 4F we see the contour lines of the stimulus phase at the time of burst onset. Their degree of dissimilarity with the contour lines of *n* is 0.17. This low value implies that the phase is yet a better candidate stimulus attribute, as compared to the hypothesis of the input slope. Note that we are considering a rather wide range of stimulus periods and amplitudes. Although for small amplitudes and long periods the level lines of slope and phase appear to be similar, there is a clear mismatch outside the lower right region in Figures 4E and F, where only the phase co-varies with *n*. We attribute the better performance of the phase code to its broader range of validity.

**Figure 5:**
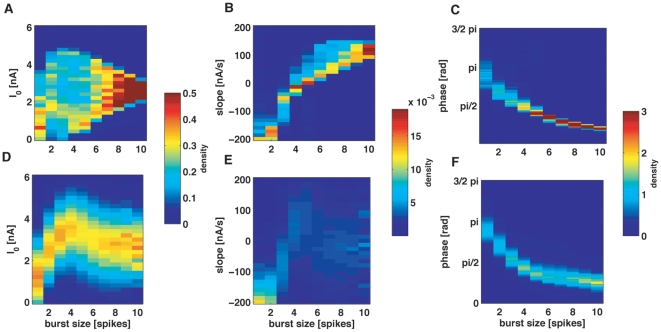
Comparison of different candidate burst-codes. (A, B, C) Normalized histograms of the stimulus amplitude (A), slope (B) and phase (C) at burst initiation, for different burst lengths *n*. For each vale of *n*, the color plot represents an estimation of the probability of each stimulus feature. The highest discriminability is obtained for the stimulus phase, given that the phase distributions show minimal overlap for different *n* values. The plots pool together all the phases obtained with the collection of stimulus amplitudes and periods used in Figure 3 B. (D, E, F) Same distributions as in A, B, C, but when the stimulus consists of a low-pass filtered Gaussian signal, with 30 Hz cut-off frequency. As for the sinusoidal case, the phase code has the maximal discriminability.

In order for a neural code based on the number of spikes per burst to be useful, the mapping between *n* and the encoded stimulus feature must not depend strongly on the properties of the input signal. For instance, changing the stimulus frequency should not lead to a significantly different mapping. We therefore assessed whether the three neural codes discussed in Figure 4 were stable with respect to changes in the stimulus amplitude and frequency. In Figure 5A–C we show the probability distribution of each candidate stimulus feature, given that the cell generated a burst of *n* spikes. Each distribution pools together the results obtained with stimuli covering a broad range of periods and amplitudes (the range is shown in Figures 4B and 4D–F). Light colors represent high probability. When considering the distribution of stimulus amplitudes (A) and slopes (B), the probability densities corresponding to different *n* values overlap significantly. Hence, by reading out the number of spikes per burst, it is not possible to guess the value of the stimulus amplitude or slope. In contrast, the correspondence between stimulus phase and *n* is remarkably narrow (Figure 5C). Therefore, all stimuli seem to induce the same mapping between phase and *n*, irrespective of the amplitude or frequency of the input signal.

### Stochastic stimulation

To test whether the results of the previous section also hold for stochastic time-varying stimuli, we explored the correspondence between input phase and *n* also for random input currents, as for example, low-passed filtered Gaussian noise of several cut-off frequencies. In the context of stochastic stimulation, the concept of phase needs to be broadened, in order to be also applicable to non-harmonic stimuli (see Methods and supporting Text S3). Any smooth time-dependent signal is the sum of a constant term and a zero-mean function. By means of the Hilbert transform (see Methods) the latter can be uniquely decomposed as the product of a positive function (the *strength* of the stimulus), and an oscillatory term whose modulus is always less or equal to one. The oscillatory term fluctuates between positive and negative values, so it can be interpreted as the sine of a time-dependent function: the *phase* of the original signal. The phase, therefore, is an angle varying between 0 and 2π. This angle represents the temporal properties of the input signal: The faster the phase grows, the higher the frequency content of the signal. If the signal is a pure sinusoid (supporting Animation S1), then the phase increases linearly in time, and its slope is proportional to the frequency. Supporting Animation S2 and Animation S3 show examples of the phase determined for amplitude and frequency modulated signals, respectively. If the input current is irregular, the phase has a complex temporal structure, as seen in the example movie of the supporting Animation S4.

In Figure 5D–F we show the probability distribution of the three candidate stimulus features, for each fixed value of *n*. Once again, when considering either the amplitude (D) or the slope (E) of the stimulus, the distributions corresponding to different *n* values overlap significantly. Hence, in agreement with the result found with periodic stimuli, stochastic stimuli confirm that *n* is not a good predictor of the stimulus amplitude or slope. In contrast, the value of the stimulus phase (ϕ) can be easily predicted from *n*. Notice that the distribution of phases at burst onset corresponding to Gaussian stimuli is remarkably similar to the one obtained with sinusoidal signals. The mapping between stimulus phase and *n* is therefore stable, irrespective of the nature of the driving signal.

One key question is how the intrinsic time scales of the neuron relate to the time scales of the stimulus to allow coding. For instance, if the stimulus time scales are much faster than the characteristic times of the neural response, then coding becomes difficult since the neuron may not be able to adjust its response at the same pace as the stimulus varies. We therefore compared the characteristic frequencies of the neuron responses with the frequency content of the cortical LFP, extending up to the limit of the high-gamma band around 150 Hz. We verified that for moderate or fast input signals, the power spectrum of the neuron membrane potential shows a characteristic band-pass profile that extends from a few Hertz to around 40 Hz, with only a weak dependence on the stimulus cut-off frequency (see Supporting Figure S3). This suggests that stimuli above 40–50 Hz may start to be sensed as too fast by the cortical neuron. In the opposite limit, that is for constant stimuli, the phase is not defined. In Figure 2 we showed that in this case the number of spikes per burst shows a weak dependence on stimulus amplitude. Therefore, we expect that for very slow stimuli the neural code undergoes a crossover to an amplitude code.

**Figure 6:**
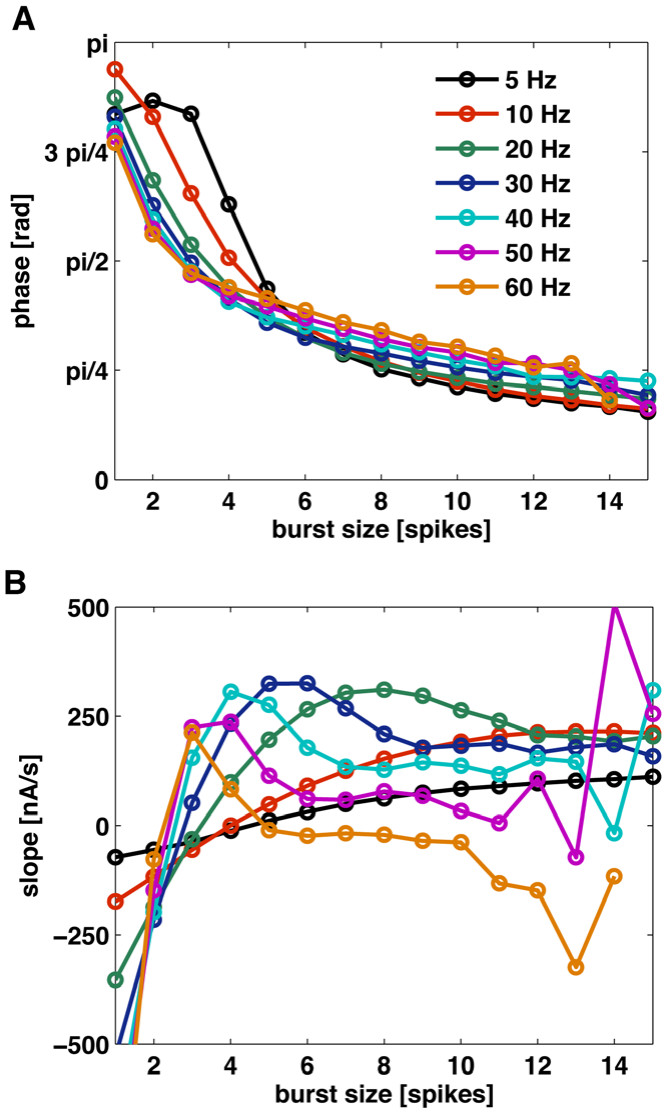
Mean values of input phase and slope as a function of burst-size. (A) Mean phase of the low-pass Gaussian stimulus at burst onset, as a function of the number of spikes per burst *n*. Different curves correspond to stimuli of different cut-off frequency. All curves collapse, indicating that all cut-off frequencies induce the same mapping between *n* and phase. The standard deviation of the stimulus is 3.6 nA. (B) Slope at burst onset for the same stimuli as in A. Different cut-off frequencies induce different mappings between *n* and slope.

With this idea in mind, we tested the validity and robustness of the phase code for stimuli with cut-off frequencies between 5 and 60 Hz. In Figure 6A, the mean value of the phase at burst onset is displayed as a function of *n*, each curve corresponding to a different cut-off frequency. The different curves almost coincide, with only a small deviation for small cut-off frequencies and for small burst sizes.

The mapping between *n* and phase is therefore almost independent of the stimulus parameters, implying that by reading out the number of spikes per burst, downstream neurons can estimate the stimulus phase independently of the context in which the bursts are fired. For comparison, in panel B we show the correspondence between stimulus slope at burst onset and *n*. Clearly, the mapping between these two quantities is much more dependent on the stimulus statistics. Although for cut-off frequencies of 5 Hz and 10 Hz the number of spikes per burst varies monotonically with the slope of the input, the correspondence between these two quantities is strongly dependent on the cut-off frequency. Therefore, unless the frequency content of the stimulus remains fixed, or downstream neurons receive parallel information about the cut-off frequency of the input signal, the slope at burst onset cannot be decoded unambiguously by reading the number of spikes per burst. For higher cut-off frequencies the relationship between burst size and input slope becomes non-monotonic, and decoding of the slope by reading the size of bursts becomes ambiguous even for a fixed cut-off frequency.

**Figure 7:**
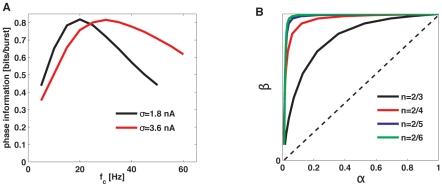
Quantification of the relationship between burst length and input phase. (A) Shannon mutual information between the stimulus phase at burst onset and the number of spikes per burst *n*. The stimulus is low-pass filtered Gaussian noise with varying cut-off frequency (horizontal axis). Different curves correspond to different stimulus standard deviations. (B) ROC curves for pairwise comparisons, for the same stimulus as in A. Different curves correspond to comparisons between different *n* values.

Having verified that there is a robust relationship between phase and *n*, one can assess their degree of correspondence using quantitative methods. To that end, we estimated the amount of phase information (see supporting Text S4) encoded in the number of spikes per burst, as shown in Figure 7. This measure quantifies the performance of a decoder that tries to guess the value of the input phase by reading out the number of spikes per burst, on a single-trial basis.

Consistent with the characteristic frequencies found in the neural responses (Supporting Figure S2), the information about input phase conveyed by the size of bursts exhibits an optimal value for stimuli with a cut-off frequency of around 20–40 Hz. Thus, bursting neurons can transmit up to 0.8 bits of phase information per burst. Given that almost all bursts are generated along the first semi-cycle of the phases between 0 and π, *n* encodes input phases with a precision of approximately 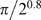. This result is in agreement with previous experimental studies: through information-theoretical measures, Montemurro *et al.* [9] showed that in visual cortex, the precision of the relative timing between spikes and the phase of the LFP is approximately π/2.

An alternative measure of the degree of correspondence between the intra-burst spike count and input phases is given by receiving operating characteristics (ROC) curves (see supporting Text S4). In Figure 7B, these curves are shown for discriminations between *n* = 2 (doublets of spikes) and several other *n* values (one curve per value). Our results show that distinguishing between different *n* values clearly allows a linear decoder to discern between different phase intervals.

**Figure 8:**
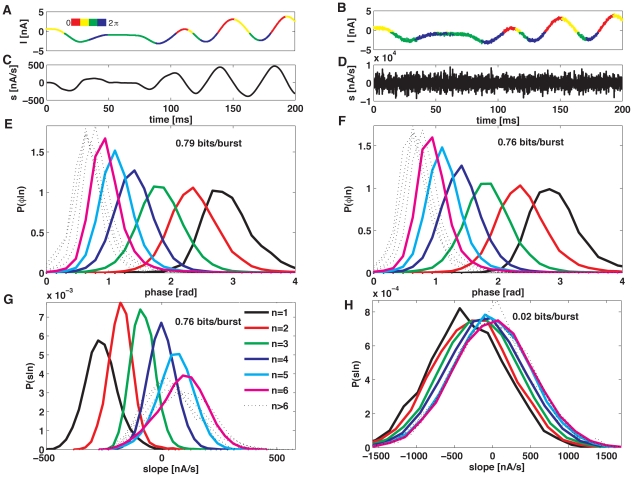
Robustness of the burst-mediated phase code. (A) Example of input stimulus (low-pass filtered Gaussian noise, with 10 Hz cut-off frequency) as a function of time. Colors represent the stimulus phases. (B) Same stimulus as in A, but with an additional high-frequency component, whose standard deviation is 10 times smaller than the standard deviation of the stimulus in A. (C) and (D) Derivative of the stimuli shown in A and B. (E) and (F) Phase probability distribution at burst onset, for the stimuli as in A and B. The high-frequency component has a minimal effect in the phase distributions. (G) and (H) Slope probability distributions for the stimuli shown in A and B. The high-frequency component has a drastic effect in the slope distributions. All curves collapse, so *n* is no longer useful to discriminate slopes.

To assess the robustness of the code, in Figure 8E and G we show the conditional distributions for phase 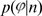 and slope 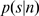, obtained with the stimulus shown in Figure 8A. In the right panels, we see how these distributions change, when a small high-frequency component is added (see panels B, F and H). The phase-conditional distributions are only slightly modified, whereas the slope distributions change radically, making slope discrimination impossible. Accordingly, the information about the phase is almost unaffected, whereas the slope information falls from 0.76 to 0.02 bits/burst. As a consequence, the slope code deteriorates rapidly, as soon as the input stimulus contains high-frequency components.

In our discussion so far, we have assumed that the slope is defined locally, in the same way as it was done by Kepecs *et al* [33]. Another way of defining the slope that takes into account intrinsic neuronal integration times would be as an average quantity integrated over a certain time window. We have repeated the analysis shown in Figure 8 using the mean slope defined as the average of the local slope over a time window of 10 ms and 20 ms. In the absence of noise, the information about the mean was substantially lower: from the 0.76 bits/burst obtained for the local definition of the slope the information decreased to 0.47 bits/burst for an integration window of 10 ms, and 0.22 bits/burst for a window of 20 ms. These values are further reduced when noise is added to the signal.

We have also verified that the information about the phase of the input signal remains high even for significantly larger levels of noise. For instance, repeating the information estimation shown in Figure 8 using noise with a standard deviation 2.5 times higher, the information about the phase is reduced by about 15%. These findings are in good agreement with recent experiments in the monkey auditory cortex [10] that have confirmed that the phase of the LFP remains a robust response feature, also in the presence of high input noise.

## Discussion

There is increasing evidence that a large amount of extra information about external stimuli can be encoded in cortical spike trains if spike timing is measured relative to the phase of the LFP. That suggests that the LFP may be used as a local clock in cortex, allowing neural populations to synchronize in small neighborhoods, and to coherently interact with other brain regions.

The feature of the LFP that most effectively can determine the pace of a local clock is its time-dependent phase. The main motivation for our work was to identify a mechanism that allowed cortical neurons to encode the phase relationship between their individual firing and the LFP, so as to make this information available to downstream neurons in an easily decodable way. To that end, we stimulated a bursting pyramidal model neuron driven by a time-dependent stimulus. The stimulus represented the total synaptic input on the neuron. Here, we assumed a close relationship between an effective time-dependent input current driving each pyramidal cell in the area and the LFP. Fluctuations in the LFP imply that the extracellular milieu varies in time. Ionic channels and pumps take the extracellular medium as the reference with respect to which they measure trans-membrane voltages. Hence, the net effect of a positive deflection in the LFP is equivalent to a negative input current into each cortical cell. In this context, the stimulus *I(t)* used in this study should be interpreted as proportional to the negative LFP. In addition, large fluctuations in the LFP have been hypothesized to co-vary with synchronous, coherent input into the cortical area [25]–[27]. To represent such coherent input, the signal *I(t)* that we have used throughout this paper can also be interpreted as a direct synaptic input into each cell. The sign of the input should depend on the type of synapses involved (excitatory or inhibitory). The two effects combined (that is, the fluctuations in the extracellular medium and the coherent synaptic input) give rise to an effective driving current *I(t)* that is proportional to the LFP [27].

In this context, we have shown that there is a tight and robust correspondence between the number of spikes per burst and the phase of the stimulus at burst onset. In the case of sinusoidal stimuli, bursting neurons lock to the driving signal. The phase of the locking depends on both the amplitude and the period of the external current. Large amplitudes and periods induce early locking, whereas small amplitudes and periods give rise to late bursting (for a possible mechanistic explanation for this effect see supporting Text S2, and supporting Figure S2). In the case of random stimuli, the signal no longer has a unique amplitude and period. However, *n* still represents the stimulus phase, and this phase is related to the local stimulus oscillation preceding burst generation.

### Experimental evidence of the robustness of the phase code

The phase code is robust with respect to stimulus statistics. The mapping between *n* and stimulus phase remains roughly invariant throughout a broad range of cut-off frequencies (see supporting Figure S4), and is not easily perturbed by input noise (Figure 8). This constancy is presumably relevant to sensory processing. Recently, Kayser *et al.* [10] have shown experimental evidence that phase codes are strikingly robust in the presence of input noise. The information in the timing of single-cell spiking (as registered with an external clock) degraded rapidly when the input stimulus was contaminated by noise. However, the information in the relative timing between single spikes and the phase of the LFP (that is, with respect to the internal clock) was only weakly affected.

### Do bursts detect input phase, input slope, or both?

Kepecs *et al.* [33] and Kepecs and Lisman [34] have shown a correspondence between *n* and input slope, claiming that the quantity encoded in burst size was the local slope. However, we have shown evidence that the variable more robustly encoded in the number of spikes per burst is the phase of the input signal. The slope code reported earlier was only valid for stimuli that included frequencies up to 20 Hz [33], and as we showed in Figure 6B that even in those cases the mapping between input slope and burst size depended strongly on the frequency cut-off of the stimulus. In fact, a slope code can simply be a consequence of the natural relationship between phase and slope for a slowly oscillating signal. As can be seen from for low-passed Gaussian white noise stimuli, these two quantities co-vary (see supporting Figure S5 A–B), albeit in a way that strongly depends on stimulus statistics. Furthermore, the slope probability distributions *P*(*s|n*) become wider for faster stimuli, whereas the phase distributions *P*(*ϕ|n*) remain essentially unchanged (see supporting Figures S4 and S5 C–D), in agreement with the idea that the neuron model is actually encoding the phase of the input signal. The relationship between phase and slope can explain the slope coding observed for the stimuli with lower cut-off frequencies in Figure 6B. However, even in that narrow range the slope code is strongly dependent on stimulus statistics. On the other hand, Figure 6A shows that the encoding of the phase is only weakly dependent on the particular frequency structure of the input signal, thus establishing a robust mapping between burst size and input phase that emerges as an intrinsic coding property of the cortical neuron.

### Encoding the full 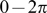 phase cycle of the input signal

In Figure 5 we showed that bursts have a strong tendency to occur on the first half cycle of an oscillating input signal; that is for phases between 0 and π. Thus, the range of encoded phases is essentially restricted to this range. However, if the net synaptic drive is projected onto pyramidal bursting neurons via inhibitory interneurons, then the effective input to the target cells is inverted in sign. In such a case, bursts are triggered in the range between 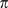 and 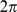 of the original input phase. Therefore, by using both populations of bursting cells (those driven by excitatory synapses and those driven via inhibitory interneurons) downstream populations should be able to decode the full phase cycle between 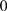 and 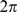. This mechanism involving two different subpopulations of bursting neurons has been suggested previously [33].

### Modulation of burst size in real neurons

Previous studies in the hippocampus [35], [36] and the olfactory bulb [37] have displayed examples where the number of spikes per burst co-varied with the phase of the input signal. We employed a theoretical model neuron to reveal the nature of this correspondence, and to provide a unifying framework for the examples previously found in these different brain areas. Mehta et al. [38] have stressed the importance of translating spike-count based codes into temporal codes, given that the latter regulate spike-timing dependent plasticity. We have taken the complementary approach: given that phase information is only available locally, we have emphasized the relevance of translating timing codes into a spike-count format that can be read out by distal target neurons. Bursting cells seem to be equipped with the intrinsic dynamic mechanisms needed for this task.

## Methods

### Pyramidal neuron model

We simulated a two-compartment conductance based model of a cortical pyramidal neuron [33], [39]–[41]. The model derives from a reduction of a multi-compartment neuron model due to Traub [42], conceived to reproduce bursting with a minimum set of ionic conductances.

The equations governing the model neuron are
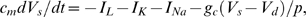
(1)for the membrane potential at the soma *V_s_*, and
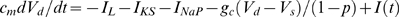
(2)for the potential in the dendritic compartment *V_d_*. The gating variables are governed by the kinetic equation
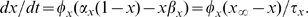
(3)The somatic sodium current reads 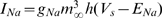, where 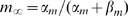, 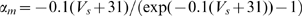, 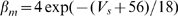, 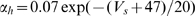, and 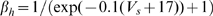. The somatic potassium current is 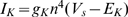, where 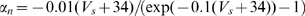 and 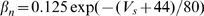.

The dendritic persistent sodium current is 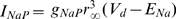, where 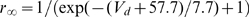. The slow potassium current is 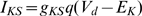, where 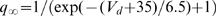 and 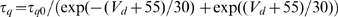. The leak currents are described by 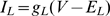, where 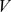 stands either 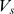 or 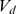, and the membrane capacitance is 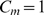 µF/cm^2^. The coupling conductance connecting the two compartments is 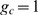 mS/cm^2^, and *p* represents the relative area between the somatic and dendritic compartments. The temperature scaling factors were 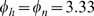. The maximum conductances were 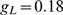, 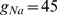, 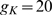 m, 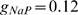, 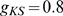, all in mS/cm^2^; while the reversal potentials were 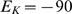, 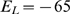, 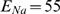, in mV. *I*(*t*) is the external input. The integration of the model was done with a 4^th^ order Runge-Kutta method using a time step of 0.01ms. We verified that neither by halving nor doubling the time step was there any change in the time evolution of the membrane potential.

#### Stimuli

We used three different types of stimuli of increasing complexity and biological realism; (a) a constant stimulus of intensity *I*, (b) a sinusoidal current described by the equation 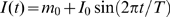, with *m_0_* = 0.6 nA; (c) a low-pass filtered Gaussian white noise with a cut-off frequency *f_c_*–this introduces a correlation time τ = 1/(2 *f_c_*). The Gaussian noise is filtered with a 4^th^ order Butterworth filter. The filtering process ensures that the signal has no frequency components above the cut-off frequency, except for a narrow range due to the filter properties. The reported standard deviations for the stimulus correspond to the final current used to drive the neuron model. When the stimulus is fully constructed, it is fed into the neural model (see Eq. (2)). In all cases explored here, the injected input current is smooth at the scale of the integration time step. Therefore, Eqs. (1–3) could be integrated by standard methods [33].

#### Relationship between synaptic input and the LFP

We assume that the total synaptic input to the neurons is proportional to the LFP [27]. In order to translate collective field potentials into the input current entering into one particular cell, synaptic time constants should be taken into account. For example, synaptic integration is often represented as a convolution of the presynaptic signal with an alpha function whose time constant is in the order of a few milliseconds. However, the frequency bands of LFPs that participate in phase-mediated neural codes lie in the delta (olfactory bulb, [43]), theta (hippocampus [5], [6]), or delta and gamma bands (cortex [44]). The correlation times of these signals are in the order of 20 ms or more, that is, much longer than typical synaptic time constants, which thus can be neglected in the present context.

#### Phase computation

A signal 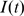 can be transformed into an analytic signal defined in the complex plane with the Hilbert transform, defined as
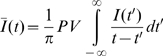
(4)Where 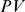 denotes the principal value of the integral [45]. The analytic representation of 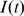 is 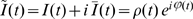. The phase of the signal is defined as 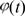, and measured between 0 and 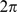. Further details can be found in Text S3, with example Animations S1, S2, S3, and S4.

#### Burst detection

Bursts were identified in simulated spikes trains as groups of spikes with an inter-spike interval (ISI) less than a predefined threshold. The threshold was determined by analyzing the ISI distribution. ISI distributions of bursting neurons have a characteristic bimodal shape, revealing two relevant times scales. At small ISIs, a sharp peak is evident, associated with the intra-burst time scale (usually less than 15ms). At longer ISIs, we find a broader peak, corresponding to the inter-burst time scale. The minimum separating the two peaks was taken as the threshold value used for burst detection [34].

**Coefficient of dissimilarity** 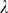 **:** We need a quantitative measure of the difference between the level maps associated with the value of different stimulus features at burst onset (Figure 3 D–F) and the level map of burst size *n* (Figure 3B) in a given domain of stimulus parameters. The index of dissimilarity 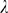 is defined as the variability of *n* in each region where the chosen stimulus feature remains confined within a certain small interval of width δ, averaged on different regions. If *n*(*y*) represents the number of spikes per burst that are elicited when the stimulus feature is equal to *y*, then the coefficient of dissimilarity is defined as 
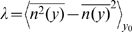
, where 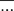 denotes an average over *y* in the infinitesimal interval (*y*_0_−δ, *y*_0_+δ) and 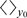 is an average over all possible values of *y*_0_ in the domain λ. When there is a perfect match between the level lines of *n* and those of the stimulus feature *y*, then λ = 0. However, when there is a mismatch, λ>0, and the dissimilarity index increases as the correspondence becomes poorer. In our analysis we always took 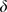 equal to 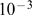 of the range of the feature *y* in the explored domain λ. The index of dissimilarity is a variance measure that quantifies the overall differences between two level maps.

## Supporting Information

Text S1 Bursting responses to constant stimuli.
(0.03 MB DOC)

Text S2 Bursting responses to sinusoidal stimuli.
(0.02 MB DOC)

Text S3 Extension of the concept of phase to non-harmonic signals.
(0.03 MB DOC)

Text S4 Quantifying selectivity.
(0.05 MB DOC)

Figure S1 Responses to sinusoidal stimuli. (A) Sample membrane potential traces (black) for two different stimuli (blue, not in scale), differing in their amplitude. For an input frequency of 40 Hz, the inter-burst period may become irregular (upper trace), or the number of spikes per burst may be variable (lower trace). For higher frequencies, locking is lost altogether, and chaotic behavior may appear. (B) Average firing rate as a function of stimulus parameters.
(0.95 MB TIF)

Figure S2 Mechanistic origin of the burst code: Relationship between the integral of the periodic stimulus over one half cycle prior to burst generation and the phase at burst onset. Different colors represent different *n* values.
(0.67 MB TIF)

Figure S3 Intrinsic time scales of the pyramidal neuron model: Power spectra of the neuron membrane potential (black) when stimulated with filtered Gaussian noise with cut-off frequencies of 2.5, 5, 20, 40, 60 and 80 Hz (from A to D). The red band indicates the range of frequencies present in the stimulus. The power spectrum of the response has a natural frequency band extending up to 40 Hz. Stimuli below 5 Hz reduce the frequency content of the response, and stimuli above 60 Hz introduce no changes in the power spectrum.
(0.09 MB EPS)

Figure S4 Selectivity of the phase and the slope codes. A–C: Probability distributions P(φ|*n*) as a function of the phase at burst onset, φ, for low-pass filtered Gaussian stimuli. Each curve represents a different number of spikes per burst *n*. D–E: Probability distributions P(*s*|*n*) as a function of the slope s. Different panels correspond to different cut-off frequencies: 10 Hz (A, D), 25 Hz (B, E) and 40 Hz (C, F). For high cut-off frequencies, the different curves are more segregated for the phase than for the slope, implying better discriminability. In addition, the slope code varies significantly as the cut-off frequency is changed (notice the expansion of the scale of slopes, in the horizontal axes of D–F).
(0.73 MB TIF)

Figure S5 Relationship between the stimulus slope and phase. (A) Scatter plot of the stimulus slope and phase, for time points chosen at random, in a Gaussian signal of 5 Hz cut-off frequency. A correspondence between the stimulus slope and phase is visible. (B) Same as A, for a Gaussian signal of 60 Hz cut-off frequency. As the cut-off frequency increases, the correspondence becomes increasingly scattered. (C) Width (measured as the standard deviation) of the probability distributions of the phase P(*φ*|*n*), for *n* = 2 and 3, as a function of the cut-off frequency. The widths remain almost constant, as the cut-off frequency increases. (D) Width of the probability distributions of the slope P(slope|*n*), as a function of the cut-off.
(0.46 MB TIF)

Animation S1 Modulus and phase of a sinusoidal signal. The actual signal (upper left panel) is taken as the real part of a complex signal. The imaginary part (second left panel) is calculated with the Hilbert transform (see Methods, main text). With these two functions, the stimulus can be interpreted as a vector that moves in the complex plane (right panel). The modulus *ρ*(t) of this vector and its phase *φ*(t) are shown in the lower left panels.
(0.03 MB GIF)

Animation S2 Modulus and phase of an amplitude-modulated signal. Same as supporting Animation S1 for an amplitude modulated signal.
(0.06 MB GIF)

Animation S3 Modulus and phase of a frequency-modulated signal. Same as supporting Animation S1 for a frequency modulated signal.
(0.74 MB GIF)

Animation S4 Modulus and phase of a low-pass filtered Gaussian signal. Same as supporting Animation S1 for Gaussian noise.
(0.04 MB GIF)
