# Supplementary material for: Conversion of Phase Information into a Spike-Count Code by Bursting Neurons
Source: PLoS One. 2010 Mar 12;5(3):e9669. doi: 10.1371/journal.pone.0009669 (PMC2837377; doi:10.1371/journal.pone.0009669)
Supplement: Text S1 — Bursting responses to constant stimuli. (0.03 MB DOC) [file pone.0009669.s001.doc]

**Bursting responses to constant stimuli**

In response to constant input currents, the membrane potential displays two different types of oscillations, operating at different time scales. On the one hand, there is a slow process of period $\Delta t$ regulating burst generation. On the other hand, there is a fast process of mean period $\delta t$, associated with spike emission in the high phases of the slow process. Depending on the strength of the input current, the cell modulates these two processes in a complex fashion. There is a current threshold, below which no spikes are observed (see Figure S3B). Above threshold, the firing rate is a monotonically increasing function of $I$, with occasional jumps. These jumps correspond to a sudden increase by one in the number of spikes per burst. Within the intervals where the number of spikes per burst remains constant, the model cell still adjusts the inter-burst period (panel D) or the intra-burst period (E), resulting in a higher firing rate for increasing input strength. Δ*t* is a continuous function of $I$, whereas *δt* tends to remain fixed at a certain value, due to refractoriness.

The four response features shown in panels B-E encode the stimulus strength, with different sensitivities. For example, the firing rate and the period $\Delta t$ are good predictors of the stimulus intensity$I$. In contrast, the period $\delta t$ and the number of spikes per burst *n* are less informative. Except for small currents, *n* remains fixed at 4 or 5 spikes per burst.
