# Supplementary material for: Conversion of Phase Information into a Spike-Count Code by Bursting Neurons
Source: PLoS One. 2010 Mar 12;5(3):e9669. doi: 10.1371/journal.pone.0009669 (PMC2837377; doi:10.1371/journal.pone.0009669)
Supplement: Text S2 — Bursting responses to sinusoidal stimuli. (0.02 MB DOC) [file pone.0009669.s002.doc]

**Bursting responses to sinusoidal stimuli**

When driven by sinusoidal stimuli, bursting responses lock to the input signal (see Figure 4A of the main text). This behavior is mainly observed in the lower frequency input signals, whereas for higher input frequencies (above 40-50Hz, depending on input amplitude) irregular responses appear, as seen in the supporting Figure S1A. In the top trace (large stimulus amplitude) the spacing between discharges is no longer regular. In the bottom trace (small amplitude) the number of spikes per burst varies between 4 and 5. As the stimulus becomes faster, increasingly irregular responses are observed.

The firing rate of the cell depends on the number of spikes per burst, the inter-burst period, and the inter-spike period inside each burst. In the supporting Figure S1B, the firing rate is displayed as a function of the maximum stimulus amplitude *I_0_* and period *T*. Inside each colored stripe of Figure 4B where *n* remains constant (see main text) the inter-burst interval varies as a function of stimulus period: as the input oscillates faster, the cell tries to keep up with the stimulus by packing subsequent bursts more closely together. When this is no longer possible, each burst looses a spike and the inter-burst frequencies increases again. As a consequence, the firing rate is a fairly complex function of the period and amplitude of the stimulus. However, the stripes of constant *n* are still visible.

As shown in the main text, the model cell anticipates firing for long bursts with respect to the input phase, and delays it for short ones. Here we present a possible explanation for this phase code. In the supporting Figure S2 we demonstrate that for periodic stimuli, the phase at burst onset co-varies with the effective input strength. We define the effective input strength as the integral of the stimulus in each semi-cycle, which is proportional to the total amount of charge that entered or left the cell, in each positive or negative oscillation. We conclude that the higher the amount of exchanged charge, the higher the spike-count and the sooner the bursting activity. The anticipation of long bursts, thus, may be explained by an increased excitability of the cell, resulting from a larger amount of input charge.
