# Supplementary material for: Conversion of Phase Information into a Spike-Count Code by Bursting Neurons
Source: PLoS One. 2010 Mar 12;5(3):e9669. doi: 10.1371/journal.pone.0009669 (PMC2837377; doi:10.1371/journal.pone.0009669)
Supplement: Text S3 — Extension of the concept of phase to non-harmonic signals. (0.03 MB DOC) [file pone.0009669.s003.doc]

**Extension of the concept of phase to non-harmonic signals**

Here we exemplify the meaning of the time-dependent phase, for several types of signals. As explained in Methods (see main text), to obtain the phase of a time-dependent stimulus we first need to analytically extend the signal to the complex plane. Thus, the original real stimulus *I*(*t*) becomes a complex signal $\tilde{I}(t)$. Graphically, one can represent the signal as a vector that moves in the complex plane, as shown in the supporting Animations S1-S4. This vector has a modulus$\rho(t)=\{\mathrm{Re}[\tilde{I}(t)]^{2}+\mathrm{Im}[\tilde{I}(t)]^{2}\}^{1/2}$, and a phase. The modulus quantifies the length of the vector, and the phase is the angle between the vector and the real axis, as seen in the bottom panels on the left of the supporting Animations S1-S4.

If the stimulus is sinusoidal (see supporting Animation S1), the real and imaginary parts of the signal are equal, and displaced in a quarter of the period. The modulus of the complex vector is constant, and the phase increases linearly in time. For an amplitude-modulated signal (supporting Animation S2), the length of the vector varies in time. The phase, however, still increases linearly. A frequency-modulated stimulus (supporting Animation S3), instead, is associated to a complex vector whose modulus is constant, whereas the phase velocity is variable. The most general case is given by an arbitrary signal, where both phase and modulus are time-varying, as in the example of the low-pass filtered Gaussian noise of the supporting Animation S4. There, both the modulus and the phase show an irregular temporal behavior.
