# Supplementary material for: Conversion of Phase Information into a Spike-Count Code by Bursting Neurons
Source: PLoS One. 2010 Mar 12;5(3):e9669. doi: 10.1371/journal.pone.0009669 (PMC2837377; doi:10.1371/journal.pone.0009669)
Supplement: Text S4 — Quantifying selectivity. (0.05 MB DOC) [file pone.0009669.s004.doc]

**Quantifying selectivity**

**Information analysis**

The statistical relationship between the burst length *n*, and the phase of the input signal at burst onset *ϕ* is given by the probability distribution *p*(*ϕ* | *n*), which can be measured from the simulated data. The unconditional distributions *p*(*ϕ*) and *p*(*n*) represent the overall distributions of phases at burst onset, and burst sizes. With these probabilities we can compute the Shannon mutual information between *ϕ* and *n*. This measure quantifies in a rigorous way the amount of information that can be obtained about the phases by counting the spikes within each burst. Shannon mutual information is defined as $I(\Phi,N)=H(\Phi)-H(\Phi|N)$[1], where the entropies are defined as

,

and

.

Using logarithms in base 2, the information is measured in units of bits. One bit of information reduces the uncertainly on a uniformly distributed variable by a factor of two. We also computed the mutual information between the burst length and the slope of the stimulus at burst onset. In this case, the information measure is based on the probability distributions $p(s|n)$ and $p(s)$, obtained from the simulated data, where *s* represents the slope of the input at the time of burst initiation. The mutual information between the slope and the burst size is $I(S,N)=H(S)-H(S|N)$.

In Figure 7A, the information between *n* and *ϕ* is depicted as a function of the cut-off frequency of the signal. For a given cut-off frequency $f_{c}\approx20-30$ Hz, the information transmitted per burst is maximal. The optimal cut-off frequency results from the fact that the burst code deteriorates for very low and very high cut-off frequencies. In the limit of $f_{c}\to0$, the stimulus varies too slowly as to resonate with the intrinsic dynamics of the cell. It is thus perceived as a constant stimulus. As demonstrated in Figure 3, constant stimuli are not well represented by the intra-burst spike count. In the opposite limit, that is, for $f_{c}\geq50$ Hz, the stimulus varies so rapidly that the amount of charge entering the cell in each upstroke (or downstroke) is small. The cell thus tends to fire short bursts, at large input phases. However, this neuron has an intrinsic tendency to burst. After several short bursts, comprising just one or two spikes, the probability to generate a long burst starts to grow, irrespective of the value of the phase. Thus, for fast stimuli, long bursts loose selectivity to the input phase (see supporting Figures S4A-C).

**ROC analysis.**

The receiving operator characteristic (ROC) curves represent the optimal performance of an ideal observer deciding on the outcome of a binary experiment in which the relevant probabilities are known [2]. In our case, we would like to know whether discriminating between bursts containing either *n_1_* or *n_2_* spikes allows us to predict whether the stimulus phase (or slope) at burst onset was higher or lower than a certain threshold *ϕ_th_*. For each threshold *ϕ_th_*, we can calculate the probability of correct guesses$p_{D}(R)$, and the related probability of false alarms$p_{F}(R)$. The plot of $p_{D}(R)$ versus $p_{F}(R)$ obtained for all possible thresholds is called the ROC curve. The diagonal corresponds to chance level. The higher the ROC curve above the diagonal the better the discriminability between the stimuli at each side of the threshold. In Figure 7B, we see the ROC curves for discriminations between *n* = 2 (doublets of spikes) and several other *n* values (one curve per value).

REFERENCES

1. Cover TM, Thomas JA (2006) Elements of information theory. New York: Wiley.
2. Green DM, Swets JA (1966) Signal detection theory and psychophysics. New York: Wiley.
