# Supplementary figures and images for: Conversion of Phase Information into a Spike-Count Code by Bursting Neurons
Source: PLoS One. 2010 Mar 12;5(3):e9669. doi: 10.1371/journal.pone.0009669 (PMC2837377; doi:10.1371/journal.pone.0009669)

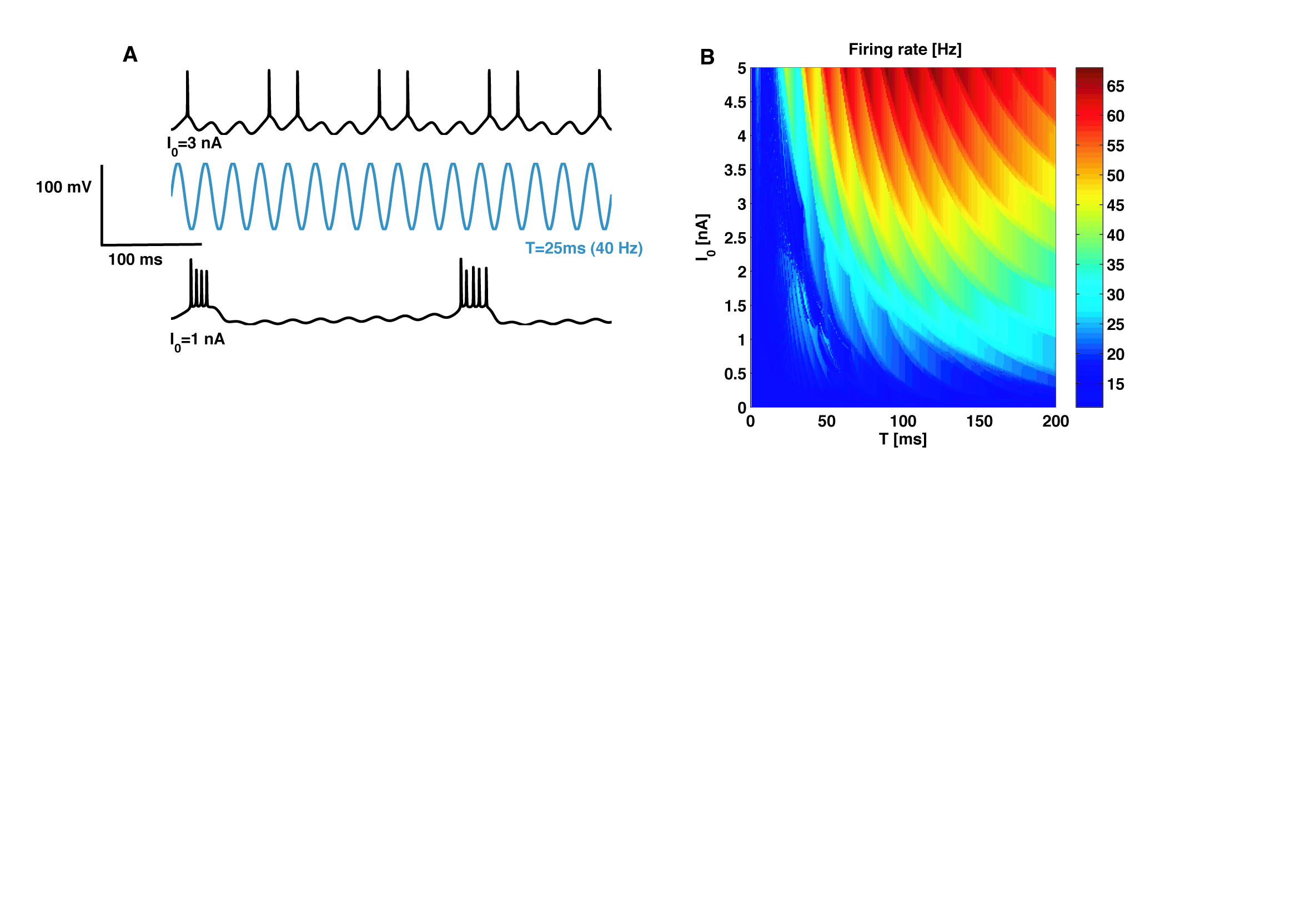

Supplement: Figure S1 — Responses to sinusoidal stimuli. (A) Sample membrane potential traces (black) for two different stimuli (blue, not in scale), differing in their amplitude. For an input frequency of 40 Hz, the inter-burst period may become irregular (upper trace), or the number of spikes per burst may be variable (lower trace). For higher frequencies, locking is lost altogether, and chaotic behavior may appear. (B) Average firing rate as a function of stimulus parameters. (0.95 MB TIF) [file pone.0009669.s005.tif]

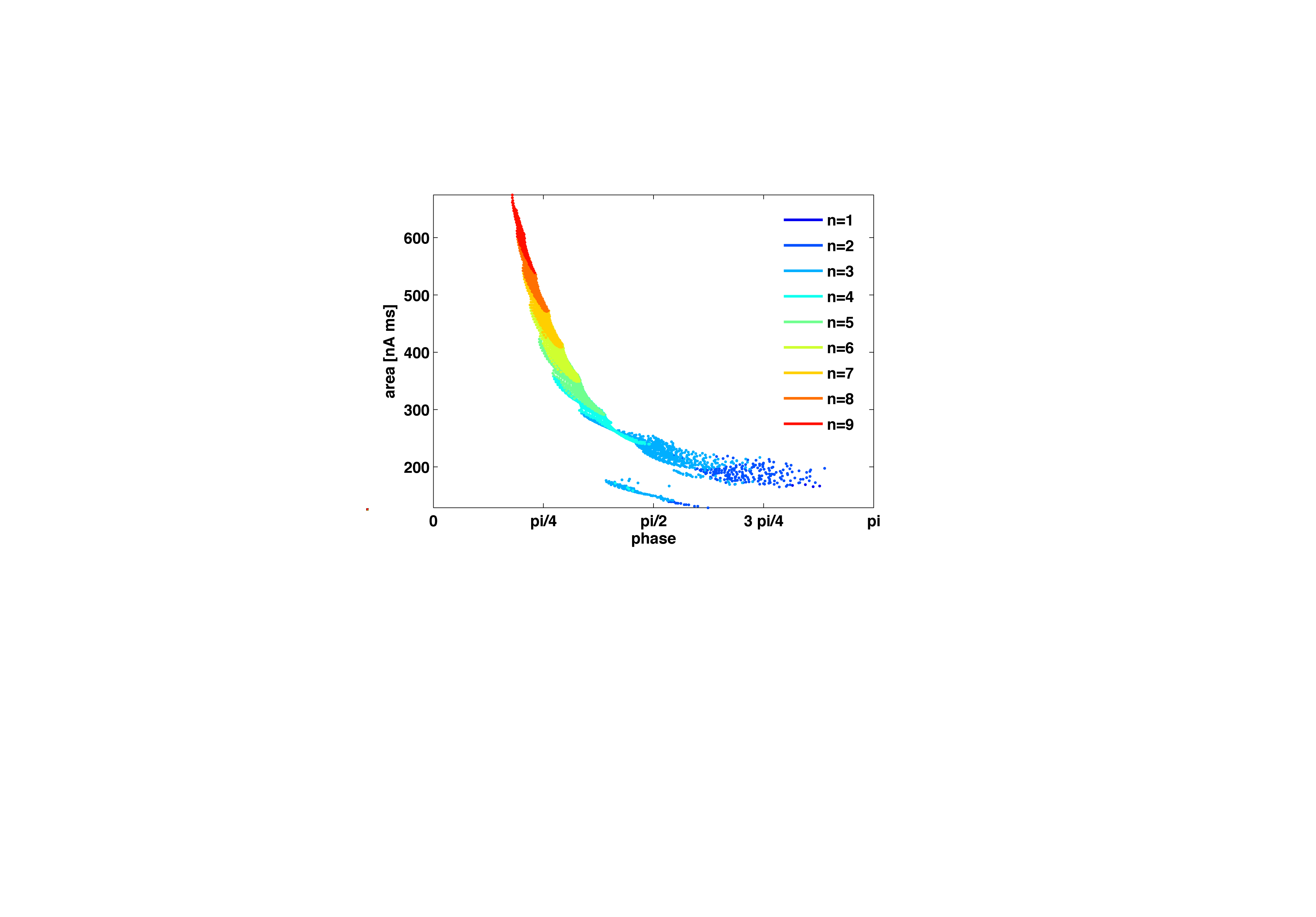

Supplement: Figure S2 — Mechanistic origin of the burst code: Relationship between the integral of the periodic stimulus over one half cycle prior to burst generation and the phase at burst onset. Different colors represent different n values. (0.67 MB TIF) [file pone.0009669.s006.tif]

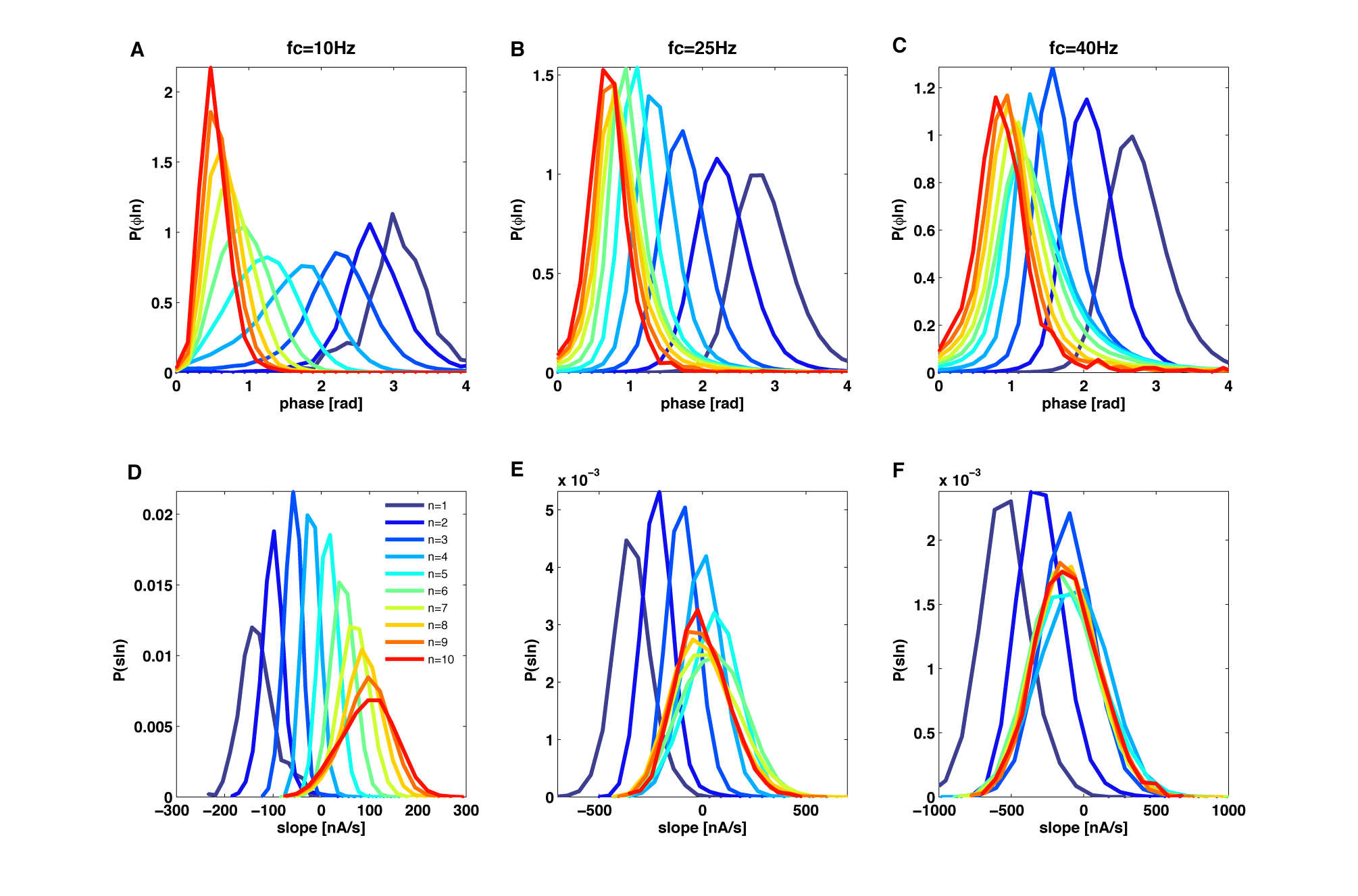

Supplement: Figure S4 — Selectivity of the phase and the slope codes. A–C: Probability distributions P(φ|n) as a function of the phase at burst onset, φ, for low-pass filtered Gaussian stimuli. Each curve represents a different number of spikes per burst n. D–E: Probability distributions P(s|n) as a function of the slope s. Different panels correspond to different cut-off frequencies: 10 Hz (A, D), 25 Hz (B, E) and 40 Hz (C, F). For high cut-off frequencies, the different curves are more segregated for the phase than for the slope, implying better discriminability. In addition, the slope code varies significantly as the cut-off frequency is changed (notice the expansion of the scale of slopes, in the horizontal axes of D–F). (0.73 MB TIF) [file pone.0009669.s008.tif]

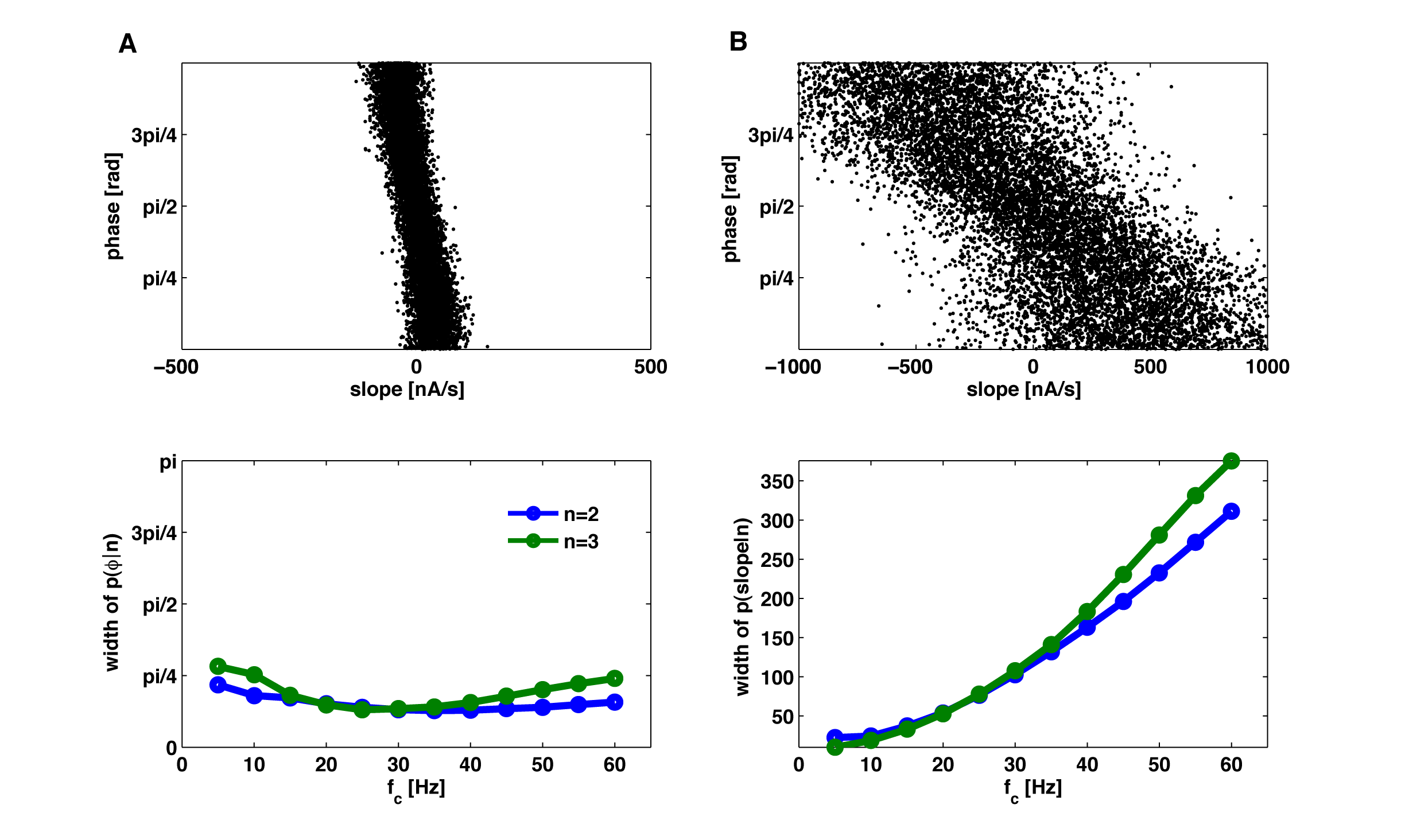

Supplement: Figure S5 — Relationship between the stimulus slope and phase. (A) Scatter plot of the stimulus slope and phase, for time points chosen at random, in a Gaussian signal of 5 Hz cut-off frequency. A correspondence between the stimulus slope and phase is visible. (B) Same as A, for a Gaussian signal of 60 Hz cut-off frequency. As the cut-off frequency increases, the correspondence becomes increasingly scattered. (C) Width (measured as the standard deviation) of the probability distributions of the phase P(φ|n), for n = 2 and 3, as a function of the cut-off frequency. The widths remain almost constant, as the cut-off frequency increases. (D) Width of the probability distributions of the slope P(slope|n), as a function of the cut-off. (0.46 MB TIF) [file pone.0009669.s009.tif]

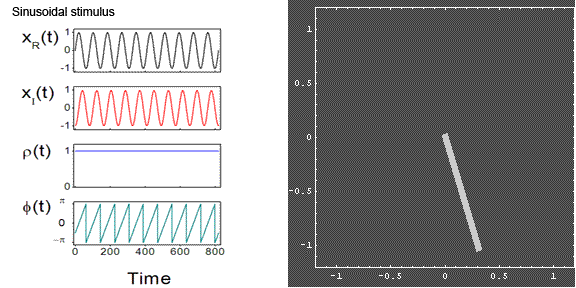

Supplement: Animation S1 — Modulus and phase of a sinusoidal signal. The actual signal (upper left panel) is taken as the real part of a complex signal. The imaginary part (second left panel) is calculated with the Hilbert transform (see Methods, main text). With these two functions, the stimulus can be interpreted as a vector that moves in the complex plane (right panel). The modulus ρ(t) of this vector and its phase φ(t) are shown in the lower left panels. (0.03 MB GIF) [file pone.0009669.s010.gif]

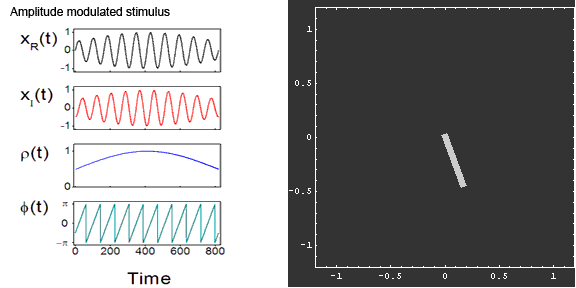

Supplement: Animation S2 — Modulus and phase of an amplitude-modulated signal. Same as supporting Animation S1 for an amplitude modulated signal. (0.06 MB GIF) [file pone.0009669.s011.gif]

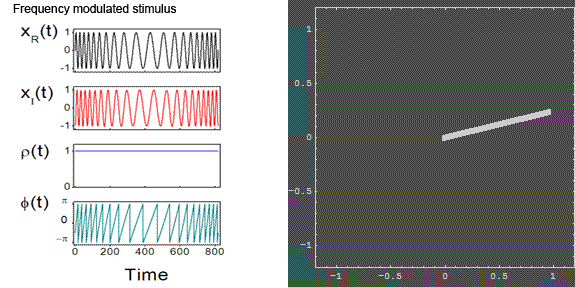

Supplement: Animation S3 — Modulus and phase of a frequency-modulated signal. Same as supporting Animation S1 for a frequency modulated signal. (0.74 MB GIF) [file pone.0009669.s012.gif]

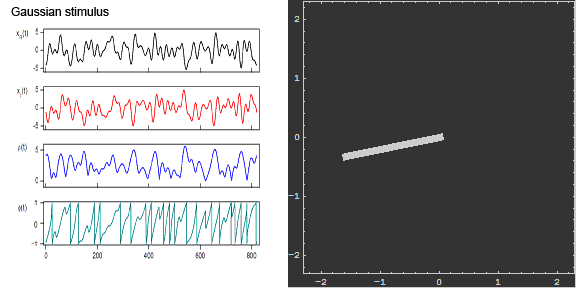

Supplement: Animation S4 — Modulus and phase of a low-pass filtered Gaussian signal. Same as supporting Animation S1 for Gaussian noise. (0.04 MB GIF) [file pone.0009669.s013.gif]
